# Integrative application of silicon and/or proline improves Sweet corn (*Zea mays* L. saccharata) production and antioxidant defense system under salt stress condition

**DOI:** 10.1038/s41598-023-45003-8

**Published:** 2023-10-25

**Authors:** Caiming Gou, Qiulan Huang, Mostafa M. Rady, Linghui Wang, Muhammad Ihtisham, Hamada H. El-awady, Mohamed Seif, Esmail M. Y. Alazizi, Rania S. M. Eid, Kuan Yan, Walid Tahri, Jia Li, El-Sayed M. Desoky, Ahmed H. El-Sappah

**Affiliations:** 1 College of Agriculture, Forestry, and Food Engineering, Yibin University, Yibin, Sichuan China; 2 Botany Department, Faculty of Agriculture, Fayoum University, Fayoum, 63514 Egypt; 3 College of Horticulture, Northwest A&F University, Xianyang, 712100 China; 4 Toxicology and Food Contaminants Department, Food Industries and Nutrition Research Institute, National Research Centre, Dokki, Giza, 12622 Egypt; 5 Sichuan University of Science and Engineering, Yibin, 643000 Sichuan China; 6 Agricultural Botany Department, Faculty of Agriculture, Benha University, Banha, 13518 Egypt; 7 International Faculty of Applied Technology, Yibin University, Yibin, 644000 Sichuan China; 8 Botany Department, Faculty of Agriculture, Zagazig University, Zagazig, 44511 Egypt; 9 Department of Genetics, Faculty of Agriculture, Zagazig University, Zagazig, 44511 Egypt

**Keywords:** Plant sciences, Environmental sciences

## Abstract

Silicon (Si) and/or proline (Pro) are natural supplements that are considered to induce plants' stress tolerance against various abiotic stresses. Sweet corn (*Zea mays* L. saccharata) production is severely afflicted by salinity stress. Therefore, two field tests were conducted to evaluate the potential effects of Si and/or Pro (6mM) used as seed soaking (SS) and/or foliar spray (FS) on Sweet corn plant growth and yield, physio-biochemical attributes, and antioxidant defense systems grown in a saline (EC = 7.14dS m^−1^) soil. The Si and/or Pro significantly increased growth and yield, photosynthetic pigments, free proline, total soluble sugars (TSS), K^+^/Na^+^ratios, relative water content (RWC), membrane stability index (MSI), α-Tocopherol (α-TOC), Ascorbate (AsA), glutathione (GSH), enzymatic antioxidants activities and other anatomical features as compared to controls. In contrast, electrolytes, such as SS and/or FS under salt stress compared to controls (SS and FS using tap water) were significantly decreased. The best results were obtained when SS was combined with FS via Si or Pro. These alterations are brought about by the exogenous application of Si and/or Pro rendering these elements potentially useful in aiding sweet corn plants to acclimate successfully to saline soil.

## Introduction

Salinity stress is a serious environmental problem that might affect agricultural output either directly or indirectly. Numerous plants cannot survive at low salinity because they are salt-sensitive. Low-quality irrigation water significantly increases the amount of dissolved salts in the soil, which reduces metabolism and shortens root shoots, inhibiting plant development and physiological processes^1^. Plant metabolism is constrained by salinity, which also lowers plants' capacity to profit from water. This affects plant growth and productivity.^2^. By generating more reactive oxygen species (ROS) such as O_2_^⋅−^, OH, and H_2_O_2_, which can alter plant metabolism due to osmotic stress and ion toxicity, salt also slows down plant development^3–6^. Reactive oxygen species are referred to be "by-products" of normal cellular metabolism when plants are subjected to salt stress and are required for the response of enzymatic inter- and intracellular coding^7^. Three effects of saline stress on plants include (1) a reduction in soil water content, which results in water shortages, (2) a specific adverse effect of Cl^−^ and Na^+^ ions; and (3) a nutritional disproportion brought on by a reduction in the absorption of specific elements^8^. Ionic and osmotic stress caused by salt stress have a direct influence on plant physiology. It modifies the relationship between plants and water, which may lead to osmotic stress or drought physiology^2,9^. In the leaf apoplast, toxins and salt buildups encourage turgor loss and dehydration, which lead to tissue and cell death. The largest physiological impact of plant salt stress on photosynthesis is attributed to the reduction of chlorophyll pigment and stomata closure that lower CO_2_ pressure^10,11^ and inhibit rubisco^12,13^.

In terms of overall productivity and area planted, maize is a spectacular cereal crop that comes in second to wheat globally^14^. *Zea mays* L. saccharata, also known as sweet corn, is a hybridized type of corn that has been cultivated to contain more sugar. Around the world, sweet corn has a significant direct and indirect role in supplying humans with calories, protein, and several vitamins and minerals. Salinity has a detrimental effect on the production of maize, which is a susceptible crop to salt stress^15^. Therefore, in order to enhance plant growth and productivity, strategies for raising maize's tolerance to salinity must be developed^16,17^. The most effective method is to develop salt-tolerant maize hybrids, but doing so requires full knowledge of the mechanisms behind maize's reactions to salt stress. However, creating salt-tolerant hybrids requires a large investment in labor, time, and investment^18^. Consequently, certain methods, including the usage of osmoprotectant materials, can be helpful in this field^19^.

To boost stress tolerance, plant osmoprotectants can be added^20^. According to Yang et al.^21^ and Ihtisham et al.^22^, one of them, Proline (Pro), can be utilized to minimize the negative consequences of oxidative stress on plants. Plant growth is regulated by signaling molecules that start signaling cascades. Additionally adaptable is the amino acid^23^. Because they protect against acute ROS impacts^24^, plants that accumulate Pro are more resistant to the detrimental effects of stress^25^. It helps most plants because of its vital physics-mechanical functions. Increasing stress tolerance in plants helps to reduce the impacts of salt stress even while Si precipitation on cell walls stimulates the majority of physiological activities^26,27^. It lessens nutrient imbalances, lowers element toxicity, and increases photosynthetic activity^28^. To lessen ion toxicity, it also raises the K^+^/Na^+^ ratio and reduces Na^+^ uptake in plants under salt stress^29^. In order to sustain healthy development in the face of Na^+^ stress, Si may also affect Na^+^ transport and distribution in various plant tissues^30^.

In this work, exogenous silicon and/or proline were administered to maize plants under salt stress (EC = 7.14 dS m^−1^), and the effects on growth and yield, physio-biochemical components, and antioxidant defense mechanisms were examined. Increases in nutrient and osmoprotectant levels, as well as antioxidants' enzymatic and non-enzymatic activities that are important for reducing salt stress, are predicted to encourage plant yield and development, MSI, RWC, and leaf anatomical features when silicon and/or proline are exogenously applied, particularly seed soaking followed by a foliar spray.

## Materials and methods

### Experimental site and plant material

Two field tests were conducted in Yibin University's greenhouse over the course of two successive growing seasons (2021 and 2022). Before each agricultural season, soil samples from the research site were collected, as shown in Table 1, and evaluated in accordance with Black et al.^31^ and Jackson^32^. Dahnke and Whitney^33^ classified the soil's EC values as mildly saline. 0.1 percent HgCl_2_ and deionized water were used to surface sterilize the grains.

**Table 1:** Physical and chemical properties of the investigated soil.

Soil characteristic	Value	
	1st season	2nd season
Soil particles distribution		
Sand, %	45.43	45.66
Silt, %	30.11	29.89
Clay, %	24.55	24.45
Textural class	Loam	Loam
Field capacity, %	16.7	16.7
CaCO_3_ (g kg^−1^)	60.4	62.0
Organic matter (g kg^−1^)	7.65	7.98
pH*	7.62	7.71
EC (dS m^−1^)**	7.11	7.17
Soluble cations and anions (mmol_c_ l^−1^)**		
Ca^2+^	16.6	16.7
Mg^2+^	19.0	19.7
Na^+^	18.1	18.0
K^+^	6.69	6.47
CO_3_^2−^	–	–
HCO_3_^−^	20.9	20.7
Cl^–^	31.7	32.7
SO_4_^2−^	8.19	8.4
Available nutrient (mg kg^−1^ soil)		
N	58.0	58.4
P	8.80	8.90
K	97.7	99.0

*Soil paste.

**Soil paste extract.

According to the best time for maize growth in the area, sweet corn (*Zea mays* L. saccharata) seed was sown on May 1 in both seasons. The seeds were cleansed with deionized water, rinsed with filtered water, and then sterilized for two minutes in 10 ml l^−1^ NaClO. They were then left to dry at room temperature (21 °C). Air-dried seeds were grown in plots 3.0 m long and 3.50 m wide, generating an area of 10.5 m^2^ in hills with rows 60 cm apart, after soaking in tap water (TW) and/or silicon or proline. The hills are 15–20 cm apart. The cultivation of maize in the area followed the recommended standard agronomic practices, such as drip irrigation and pest and disease control. Before sowing, 33 kg P unit of Ca (H_2_PO_4_)_2_ (15.5% P_2_O_5_) and 95 kg K unit of K_2_SO_4_ (48% K_2_O) were added to each ha. Additionally, five split applications of 300 kg N unit ((NH_4_)2SO_4_; 21% N) per ha were made seven days following seeding.

### Application or silicon and/or proline

Silicon was used at a concentration of 6 mM Si (in Stable Water-Soluble Silicic Acid Potassium Salt, Potassium Silicate powder (K_2_SiO_3_) at pH 11.3; Code 1312-76-1 at Henan Daken Chemical Co., LTD, China) and proline was used at a concentration of 6 mM Pro (Pure L-Proline Powder Compliant with US Pharmacopoeia (USP) Quality Standard, NuSci Brand). Three times, at 25, 40, and 55 days following planting to run-off, silicon or proline was applied as a foliar spray (FS), with the addition of a few drops of Tween-20 as a surfactant to ensure effective and complete spray solution penetration.

### Maize Morphological Traits and yield component

After 65 days, the plants were harvested. To measure plant height (cm) and other physiological traits, ten maize plants from each treatment were removed. Samples were gathered during harvest from ten randomly selected plants to determine the number of grains per row, the number of rows per ear, the grain yield (kg/ha), the bio yield (kg/ha), and the 1000-grain weight.

### Determination of chlorophyll content, PSII quantum yield, and CO_2_ fixation rate

Avron^34^ eliminated all of the carotenoids and chlorophyll from the fresh leaf using only pure acetone. The net photosynthetic rate (Pn), rate of transpiration (Tr), and stomatal conductance (gs) of leaves were measured using a portable photosynthesis system (LF6400XTR, LI-COR, USA). Between nine in the morning and eleven in the morning, the tests were given.

### Determinations of RWC, MSI, EL, MDA, Leaf soluble sugars, and proline

Barrs and Weatherley^35^ described the relative water content (RWC), whereas Premchandra et al.^36^ created the membrane stability index (MSI). Sullivan and Ross published a technique for calculating the total ions in leafy tissue in 1979. Electrical conductivities (EC1, EC2, and EC3) of a 20-leaf tissue disc solution were determined 3 times: before heating, during a 30 min. heating period at 45–55 °C, and after ten min. of boiling at 100 °C. EL was calculated using the following well-known formula:

$${\text{EL }}\left( \% \right) \, = \, \left[ {\left( {{\text{EC2 }} - {\text{ EC1}}} \right)/{\text{EC3}}} \right] \, \times { 1}00$$

Malondialdehyde (MDA; mol/g of leaf FW) concentration was evaluated to evaluate lipid peroxidation. Utilizing the same H_2_O_2_ extracts, MDA was assessed. The MDA concentration was calculated using a coefficient of molar extinction of 0.155 10^−3^ M^−1^ cm^−1^^37^.

Irigoyen et al.^38^ procedure was used to extract and assess the total soluble sugar content. A dried leaf sample (0.2 g) was homogenized in 5 ml of 96% (v/v) ethanol before being rinsed in 5 ml of 70% (v/v) ethanol. Before measurement, centrifuging the extract at 3500*g* for 10 min. was necessary, and then the supernatant was put to store at 4 °C. Anthrone reagent, which is created by mixing 150 mg of anthrone with 100 ml of 72% (v/v) sulphuric acid, was added to 0.1 ml of the ethanolic extract in order to determine the amount of soluble sugar contained. After that, the mixture was cooked for 10 min in a bath of boiling water. After cooling, absorbances at 625 nm were measured using a Bauschand Lomb-2000 Spectronic Spectrophotometer (ThermoSpectronic, Mercers Row, Cambridge, UK).

The proline amounts in 0.5g dehydrated leaf specimen were determined using fast colorimetry and the Bates et al.^39^ technique. Following the extraction in 10 ml of 3% (v/v) sulphosalicylic acid, a 10-min. centrifugation at 10,000*g* was carried out. After the reaction was halted with a cold bath, the extraction was done with 5 ml of toluene and 15 s of vortex mixing. The toluene and aqueous phases were separated in the shadows for 20 min. at room temperature. The top toluene phase was carefully gathered and then the absorption at 520 nm was measured.

### Determinations of nutrient content

The following concentrations of nitrogen (N), phosphorus (P), potassium (K), calcium (Ca), and sodium (Na) were estimated: digested 0.2 g of dried leaf with sulphuric acid in the presence of H_2_O_2_^40^. Total Na, Ca, and K concentrations were measured directly using a Flame photometer^41^. Total N was determined using a microkjeldahl method, according to Chapman and Pratt^42^. Total P was determined calorimetrically using the ascorbic acid method^43^.

### Determinations of antioxidant enzyme activities

The enzymes were extracted using the technique described by Vitoria et al.^44^. According to Chance and Maehly^45^, Fielding and Hall^46^, Sairam et al.^47^, and Rao et al.^48^, the enzymes ascorbate peroxidase (APX), peroxidase (POD), glutathione reductase (GR), catalase (CAT), and superoxide dismutase (SOD) were assessed.

### Determination of non-enzymatic antioxidant compounds and oxidative stress (hydrogen peroxide; H_2_O_2_, and superoxide; O_2_^⋅‒^)

Kampfenkel and Van Montagu^49^ used a component of 30 mM potassium phosphate buffer pH 7.4 + 2.5% TCA + 8.4% H_2_PO_4_ + 0.8% bipyridyl + 0.3% FeCl_3_ to assess the concentration (mol g1 FW) of ascorbate asa. The reaction was run at 40 °C for 30 min while measuring the absorbance at 525 nm. Griffth^50^ reported that the amounts of total and reduced glutathione (GSH) (mol g1 FW) were measured. A reaction combination including leaf extract, 130 mM sodium phosphate buffer, pH 7.4 + 7 mM sodium phosphate buffer, pH 6.8 with 6 mM DTNB, and 5, 5-dithiobis-(2-nitrobenzoic acid) was used to measure the GSH concentration. Prior to measuring the absorbance at 412 nm, the reaction mixture was kept at 30 °C for 10 min.

The tocopherol content of each gram of leaf dry weight was calculated. The extraction solvent (n-hexane–ethyl acetate, n-hexane + 0.1 L of CH_3_–COO–CH_2_–CH_3_) included 0.9 L and 0.02 g of butylated hydroxytoluene (BHT). R-TOC was combined with 0.05 g of n-hexane in 0.1 L to create standard solutions (0.02–0.2 mg per mL). Then, an HPLC system with a phase ratio of 94 methanol /6 water, flow rate of 1.5 mL/min, and 292 smm UV detector were used to determine the α-TOC concentration^51^.

By homogenizing 0.25 g of garden-fresh leaf in 5 ml of 5% TCA, the H_2_O_2_ concentration (mol per g of leaf FW) was calculated. It took 15 min to complete a homogenate centrifugation (12,000*g*) at 4 °C. A reaction medium with 10 mM of buffer (potassium phosphate, pH 7.0) and 1 M of KI was added after the supernatant had been collected. According to Velikova et al.^52^, absorbance at 390 nm was measured using a spectrophotometer and an H_2_O_2_ standard.

In order to quantify O_2_^⋅^, 100 mg of a fresh, completely inflated maize leaf was divided into 1 mm-sized pieces. The pieces were then immersed for a further hour at room temperature in a solution of 10 mM K-phosphate buffer, pH 7.8, 0.05% NBT, and 10 mM NaN_3_. After 15 min of heating at 85 °C, a 2 ml immersed solution was instantly cooled. A580 g^-1^ FW was used as the O_2_^⋅−^ concentration when the optical density was calorimetrically measured at nm^53^.

### Anatomical studies

For comparative microscopy at 50 DAS, the main stem leaflet's median area from the 2021 (the second growing season) experiment was employed. Plants from the best (Put) treatment and the control, as well as three water levels, were used. The samples were fixed following the Paolillo and Zobel^54^ method, which calls for the use of the FAA solution (formalin, acetic acid, and alcohol). Using double-edged razor blades, the conserved leaflet and stem were cut into pieces (5 mm long), which were then cut into thin cross sections. After that, Johansen's pigments, fast green, and safranin were accustomed to coloring the cross sections^55^. The samples were cleaned with ethanol and xylene methyl salicylate (1:2, v/v) before being quickly examined under a microscope and prepared for comprehensive imaging. Using a Thermo Fisher Scientific EVOS FL Cell Imaging System, excellent images were captured.

### Statistical analysis

The study's data were analyzed using SPSS software, version 19.0. The significant changes between the investigated treatments were displayed using the Least Significant Difference (LSD) at P 0.05.

### Statement

We confirm that the plant material was collected by Yibin University guidelines and legislation. We follow the IUCN Policy Statement on Extinction-Risk Species Research and the Convention on International Trade in Endangered Species of Wild Fauna and Flora.

## Results

### Growth, yield, photosynthetic pigments, and photosynthetic efficiency

All Si and/or pro treatments performed better than the control (SS in FS + TW with TW). As an example, SS in FS + TW with Si, SS in FS + TW with Pro, SS in FS + Si with TW, SS in FS + Pro with Si, and SS in FS + TW with Pro are all used as SS or FS. Indicators of yield (such as the number of grains per row and the number of rows per ear), photosynthetic pigments (such as chlorophyll a and b, carotenoids), and photosynthetic efficiency (such as transpiration rate, net photosynthetic rate, and stomatal conductance) all rose with the height of the plant. Similar patterns are seen in the data for the 2021 and 2022 growing seasons (Table 2). The best therapy in FS + Si with Si was the integrative SS, followed by Si + FS with Pro and considerably outperforming other integrative treatments in both seasons.

**Table 2:** Seed soaking in and/or foliar spray by silicon or proline effects on growth, yield traits, chlorophyll a and b, carotenoids, net photosynthetic rate, transpiration rate, stomatal conductance of salt-stressed maize plants in two growing seasons.

Seed soaking	Foliar spray	Plant height (cm)	No. of grain/row	No of rows/ear	grain yield (kg/ha)	Bio yield (kg/ha)	1000‐grain weight
1st season							
TW	TW	181.1 ± 6.2^g^	25.2 ± 1.2^g^	11.7 ± 1.1^c^	4008 ± 9.3^g^	9185 ± 13^f^	191.8 ± 6.3^g^
TW	Si	196.8 ± 7.1^e^	29.3 ± 1.5^e^	13.1 ± 1.3^b^	4913 ± 9.8^e^	11,913 ± 16^d^	212.6 ± 6.9^e^
TW	Pro	193.1 ± 6.9^f^	27.6 ± 1.3^f^	12.8 ± 1.2^bc^	4564 ± 9.9^f^	9635 ± 15^e^	203.1 ± 7.5^f^
Si	TW	211.3 ± 8.2^c^	33.7 ± 2.1^c^	13.8 ± 1.4^b^	5501 ± 9.4^c^	13,001 ± 16^b^	226.2 ± 8.6^c^
Pro	TW	202.4 ± 8.9^d^	32.0 ± 2.6^d^	13.5 ± 1.3^b^	5085 ± 8.6^d^	12,447 ± 19^c^	218.8 ± 8.3^d^
Si	Si	227.0 ± 8.3^a^	38.4 ± 2.3^a^	16.5 ± 1.6^a^	6014 ± 10^a^	13,865 ± 15^a^	240.3 ± 8.5^a^
Pro	Pro	221.3 ± 8.5^b^	35.0 ± 2.5^b^	15.5 ± 1.7^b^	5821 ± 12^b^	13,120 ± 18^b^	233.8 ± 8.9^b^
2nd season							
TW	TW	179.7 ± 7.5^g^	25.6 ± 1.6^f^	12.8 ± 0.9^d^	4042 ± 11^e^	9205 ± 16^f^	193.2 ± 9.3^g^
TW	Si	197.8 ± 7.6^e^	30.3 ± 1.3^d^	13.3 ± 1.4^cd^	4980 ± 12^c^	11,926 ± 13^d^	213.7 ± 9.6^e^
TW	Pro	193.7 ± 6.9^g^	28.3 ± 1.6^e^	13.2 ± 1.3^cd^	4630 ± 15^d^	9651 ± 18^e^	203.8 ± 9.8^f^
Si	TW	212.3 ± 6.4^c^	34.3 ± 2.6^b^	14.2 ± 1.5^b^	5567 ± 14^b^	13,025 ± 19^b^	228.3 ± 8.9^c^
Pro	TW	203.3 ± 8.3^d^	32.6 ± 2.9^c^	13.9 ± 1.6^bc^	5152 ± 16^c^	12,483 ± 20^c^	219.4 ± 8.7^d^
Si	Si	227.7 ± 7.9^a^	39.3 ± 2.8^a^	15.8 ± 1.7^a^	6081 ± 13^a^	13,878 ± 23^a^	240.8 ± 9.3^a^
Pro	Pro	223.2 ± 8.9^b^	35.6 ± 2.3^b^	15.2 ± 1.6^a^	5888 ± 15^a^	13,143 ± 21^b^	234.8 ± 9.2^b^

Seed soaking	Foliar spray	Chl. a (mg g^−1^)	Chl. b (mg g^−1^)	Carotenoids (mg g^−1^)	Net photosynthetic rate (µmol CO_2_ m^‒2^ s^‒1^)	Transpiration rate (mMol H_2_O m^‒2^ s^‒1^)	Stomatal conductance (mMol H_2_O m^‒2^ s^‒1^)
1st season							
TW	TW	1.09 ± 0.9^g^	0.530 ± 0.3f.	0.81 ± 0.06^g^	6.46 ± 0.22^g^	3.35 ± 0.11f.	0.253 ± 0.01^g^
TW	Si	1.34 ± 0.08^e^	0.763 ± 0.5^de^	1.23 ± 0.09^e^	10.7 ± 0.69^e^	5.94 ± 0.25^d^	0.456 ± 0.03^e^
TW	Pro	1.28 ± 0.11f.	0.750 ± 0.04^e^	1.13 ± 0.08f.	9.84 ± 0.85f.	5.39 ± 0.36^e^	0.423 ± 0.02f.
Si	TW	1.49 ± 0.13^c^	0.826 ± 0.06^c^	1.30 ± 0.09^c^	12.2 ± 0.95^c^	7.03 ± 0.45^c^	0.516 ± 0.04^c^
Pro	TW	1.40 ± 0.12^d^	0.783 ± 0.06^d^	1.26 ± 0.08^d^	11.7 ± 0.99^d^	6.23 ± 0.44^d^	0.486 ± 0.03^d^
Si	Si	1.75 ± 0.14^a^	0.896 ± 0.07^a^	1.38 ± 0.07^a^	13.5 ± 0.98^a^	7.81 ± 0.68^a^	0.583 ± 0.04^a^
Pro	Pro	1.57 ± 0.13^b^	0.863 ± 0.06^b^	1.34 ± 0.09^b^	13.0 ± 0.96^b^	7.56 ± 0.65^b^	0.550 ± 0.03^b^
2nd season							
TW	TW	1.10 ± 0.08^g^	0.516 ± 0.02^e^	0.820 ± 0.04f.	6.49 ± 0.41^g^	3.40 ± 0.12^g^	0.260 ± 0.01^e^
TW	Si	1.35 ± 0.09^e^	0.772 ± 0.04^d^	1.24 ± 0.07^d^	10.8 ± 0.65f.	5.97 ± 0.33^e^	0.470 ± 0.02^c^
TW	Pro	1.29 ± 0.09f.	0.770 ± 0.06^d^	1.15 ± 0.06^e^	9.96 ± 0.96f.	5.41 ± 0.36f.	0.433 ± 0.04^d^
Si	TW	1.50 ± 0.11^c^	0.836 ± 0.07^c^	1.31 ± 0.08^c^	12.4 ± 0.98^c^	7.10 ± 0.54^c^	0.526 ± 0.03^b^
Pro	TW	1.41 ± 0.12^d^	0.793 ± 0.04^d^	1.28 ± 0.09^d^	11.7 ± 0.91^d^	6.37 ± 0.45^d^	0.503 ± 0.04^b^
Si	Si	1.76 ± 0.13^a^	0.910 ± 0.08^a^	1.39 ± 0.08^a^	13.9 ± 0.89^a^	8.02 ± 0.39^a^	0.596 ± 0.05^a^
Pro	Pro	1.59 ± 0.12^b^	0.876 ± 0.06^b^	1.35 ± 0.06^b^	13.3 ± 1.1^b^	7.59 ± 0.65^b^	0.580 ± 0.04^a^

Data are means (n = 5) ± SE. The same letters in each column indicate no significant differences according to the LSD test (p ≤ 0.05).

*TW* tap water, *Si* silicon, *Pro.* proline.

### RWC, MSI, EL, MDA, free praline, and total soluble sugars

**Figure 1:**
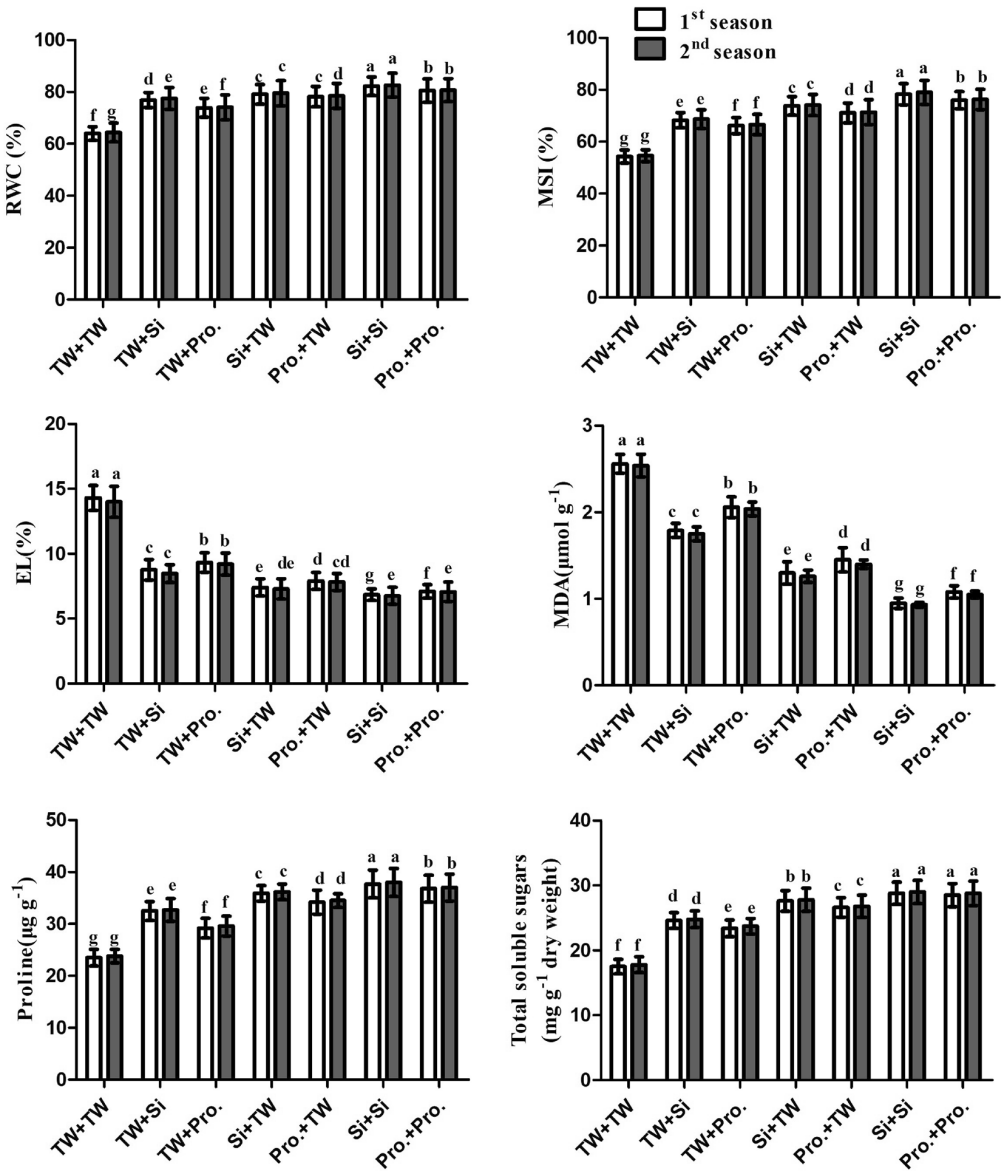
Seed soaking in and/or foliar spray by silicon or proline effects on relative water content (RWC), membrane stability index (MSI), electrolyte leakage (EL), malondialdehyde (MDA), proline content, and total soluble sugars content of salt-stressed maize plants in two growing seasons. Data are means (n = 5) ± SE. The same letters in each column indicate no significant differences according to the LSD test (p ≤ 0.05). *TW* tap water, *Si* silicon, *Pro.* proline.

The SS in FS + TW with Si, SS in FS + TW with Pro, SS in FS + Si with TW, SS in FS + Pro with TW, SS in FS + Si with Si, and SS in Fs + Pro with Pro Treatments significantly (P ≤ 0.05) increased the RWC, MSI, free proline, and soluble sugars while significantly (P ≤ 0.05) decreased MDA and EL when compared to the SS in TW + FS with TW control treatment. The statistics show parallel patterns in the growth seasons of 2021 and 2022 (Fig. 1, Supplementary Table S1).

When compared to other integrative treatments, the integrated Pro in Si + FS with Pretreatment and the integrated SS in FS + Si with Si generated the best outcomes.

### Nutrient contents and K^+^/Na^+^ ratio

**Figure 2:**
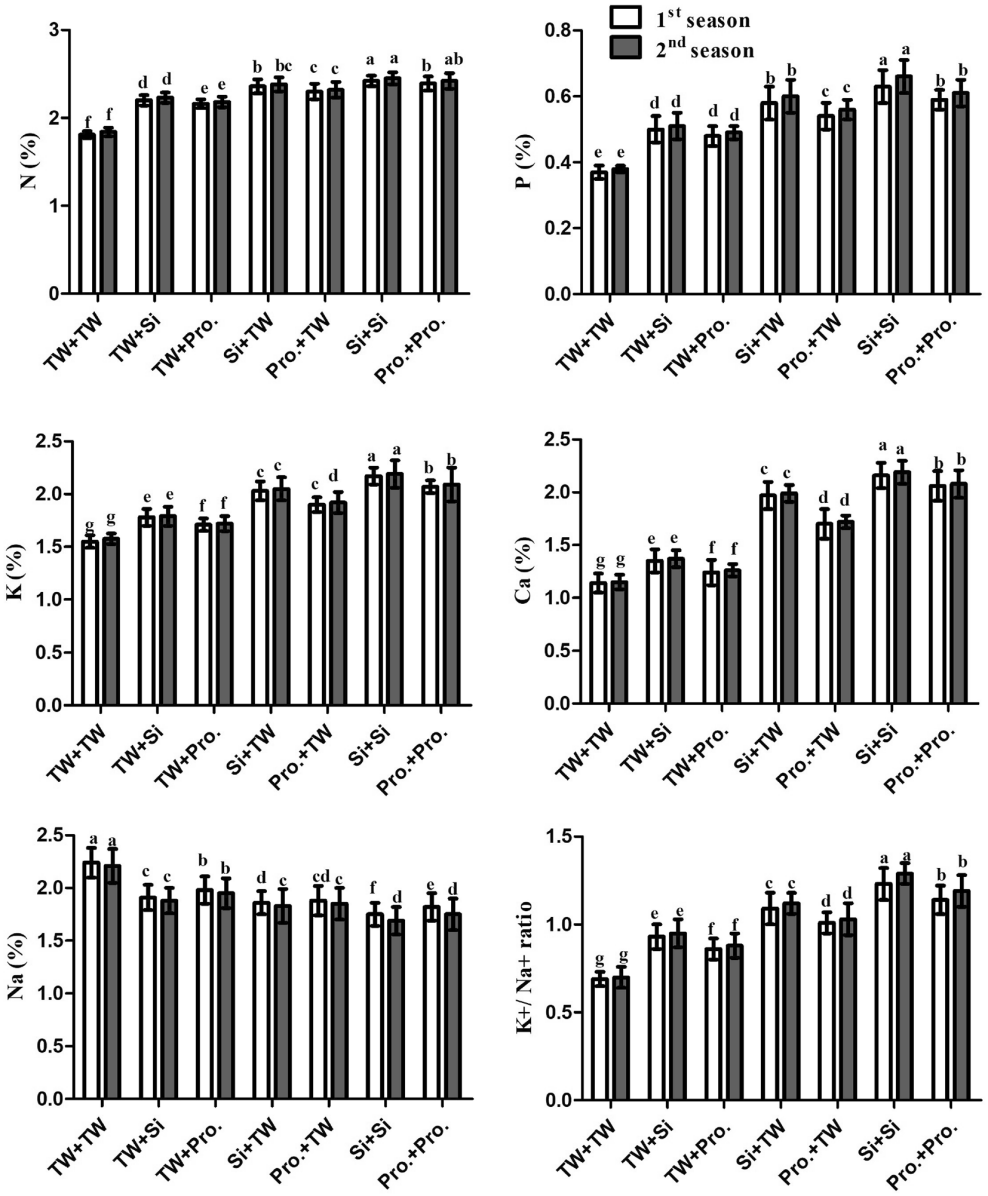
Seed soaking in and/or foliar spray by silicon or proline effects on nutrient content (i.e. N, P, K, Ca, and Na) and K^+^/Na^+^ ratio of salt-stressed maize plants in two growing seasons. Data are means (n = 5) ± SE. The same letters in each column indicate no significant differences according to the LSD test (p ≤ 0.05). *TW* tap water, *Si* silicon, *Pro.* proline.

In contrast to the control (SS in TW plus FS with TW), the SS in Si or FS + Pro with Si or Pro treatment expanded the content of N, P, Ca, and K, as well as the K^+^/Na^+^ ratio. even though much decreasing Na^+^ content (P 0.05). Results show similar patterns for the 2021 and 2022 growing seasons (Fig. 2; Table 1), demonstrating because of the integrated SS in SS in Si + FS with Si therapy was the most successful and outperformed all other integrative therapies.

### Enzymes activities (CAT, POX, APX, SOD; and GR), non-enzymatic antioxidant compounds (AsA, GSH, and α-TOC), and oxidative stress markers (H_2_O_2_, and O_2_^⋅−^).

**Figure 3:**
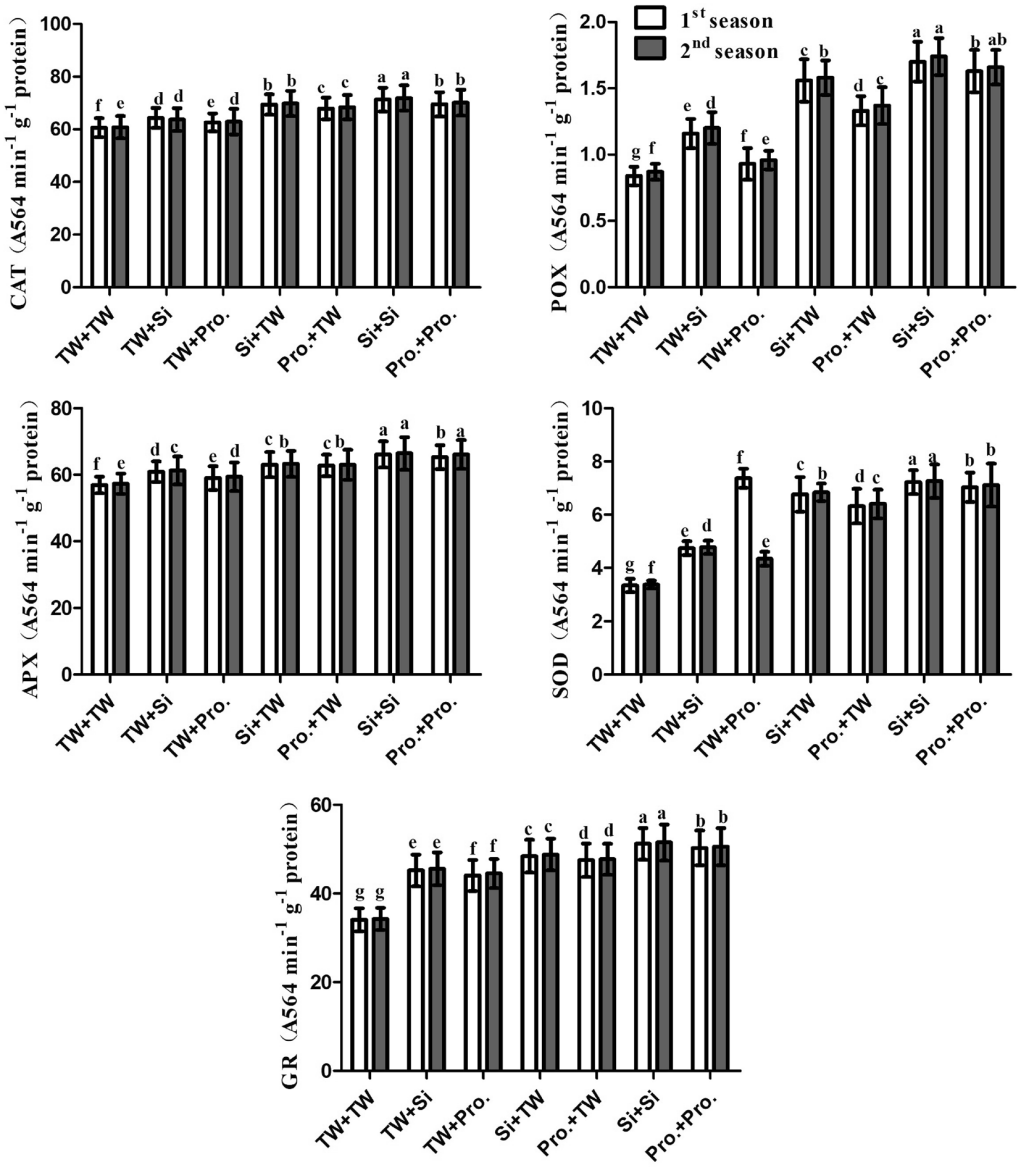
Seed soaking in and/or foliar spray by silicon or proline effects on antioxidant enzymes of salt-stressed maize in two growing seasons. Data are means (n = 5) ± SE. The same letters in each column indicate no significant differences according to the LSD test (p ≤ 0.05). *TW* tap water, *Si* silicon, *Pro.* proline.

**Figure 4:**
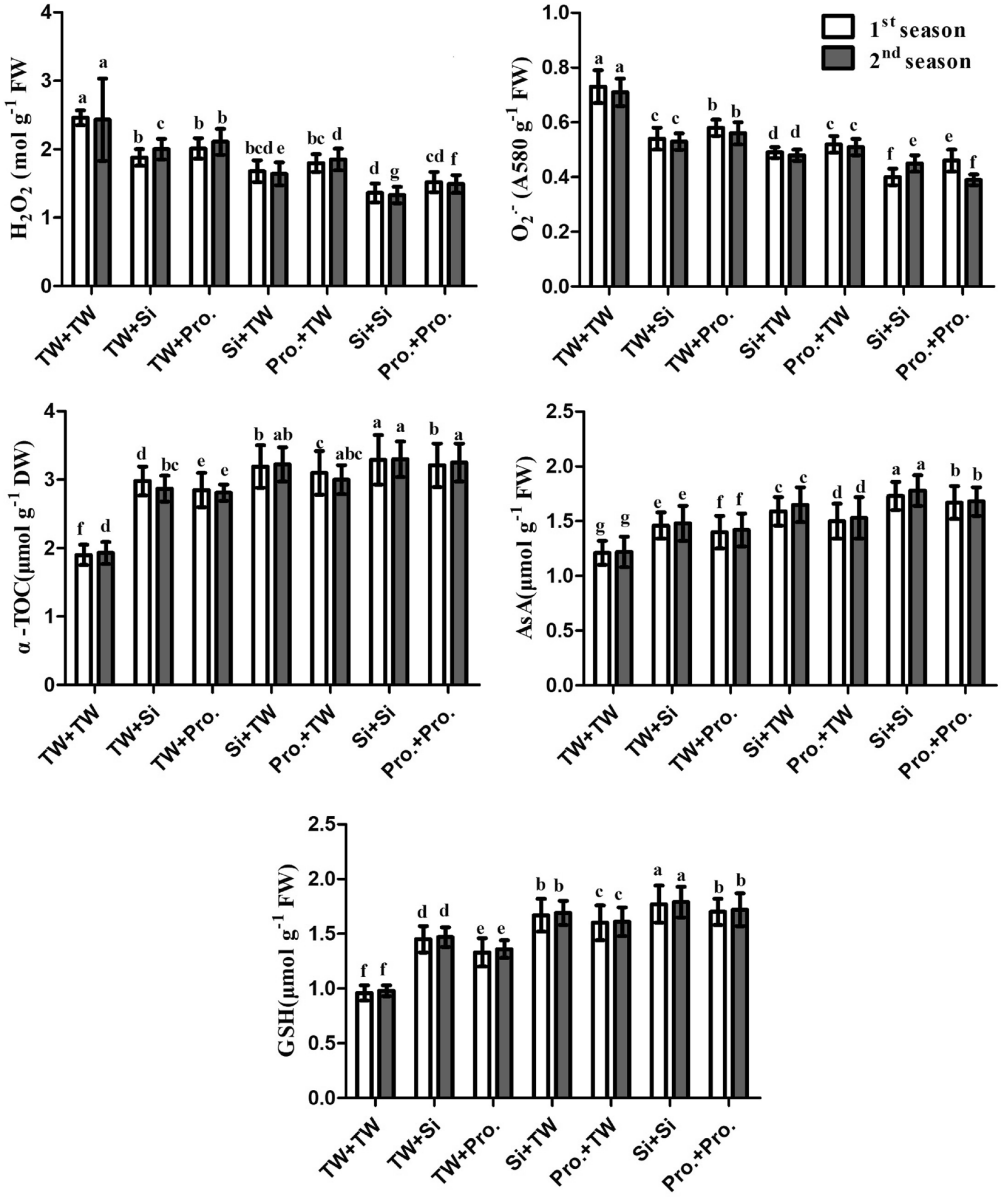
Seed soaking in and/or foliar spray by silicon or proline effects on hydrogen peroxide (H_2_O_2_), superoxide radical (O_2_^⋅−^), α-Tocopherol (α-TOC), Ascorbate (AsA) and glutathione (GSH)of salt-stressed maize plants in two growing seasons. The same letters in each column indicate no significant differences according to the LSD test (p ≤ 0.05). *TW* tap water, *Si* silicon, *Pro* proline.

In contrast to the control (SS in TW plus FS with TW), every therapy of Si and/or Pro Used as SS or FS (i.e., SS in FS + TW with Si, SS in FS + TW with Pro, SS in Si + FS with TW, SS in FS + Pro with TW, SS in Si + FS with Si and SS in FS + Pro with Pro) strongly (P ≤ 0.05) raised GR, SOD, APX, POX, CAT, AsA, GSH and α-TOC while decreased H_2_O_2_ and O_2_^⋅−^.Similar patterns are seen in the data for the 2021 and 2022 growing seasons (Figs. 3, 4, Supplementary Table S2). The integrated SS in Si + FS with Si and the integrated Pro in Si + FS with Pro, both of which outperformed other integrative therapies, were the best therapies in both seasons.

### Leaf anatomy

**Figure 5:**
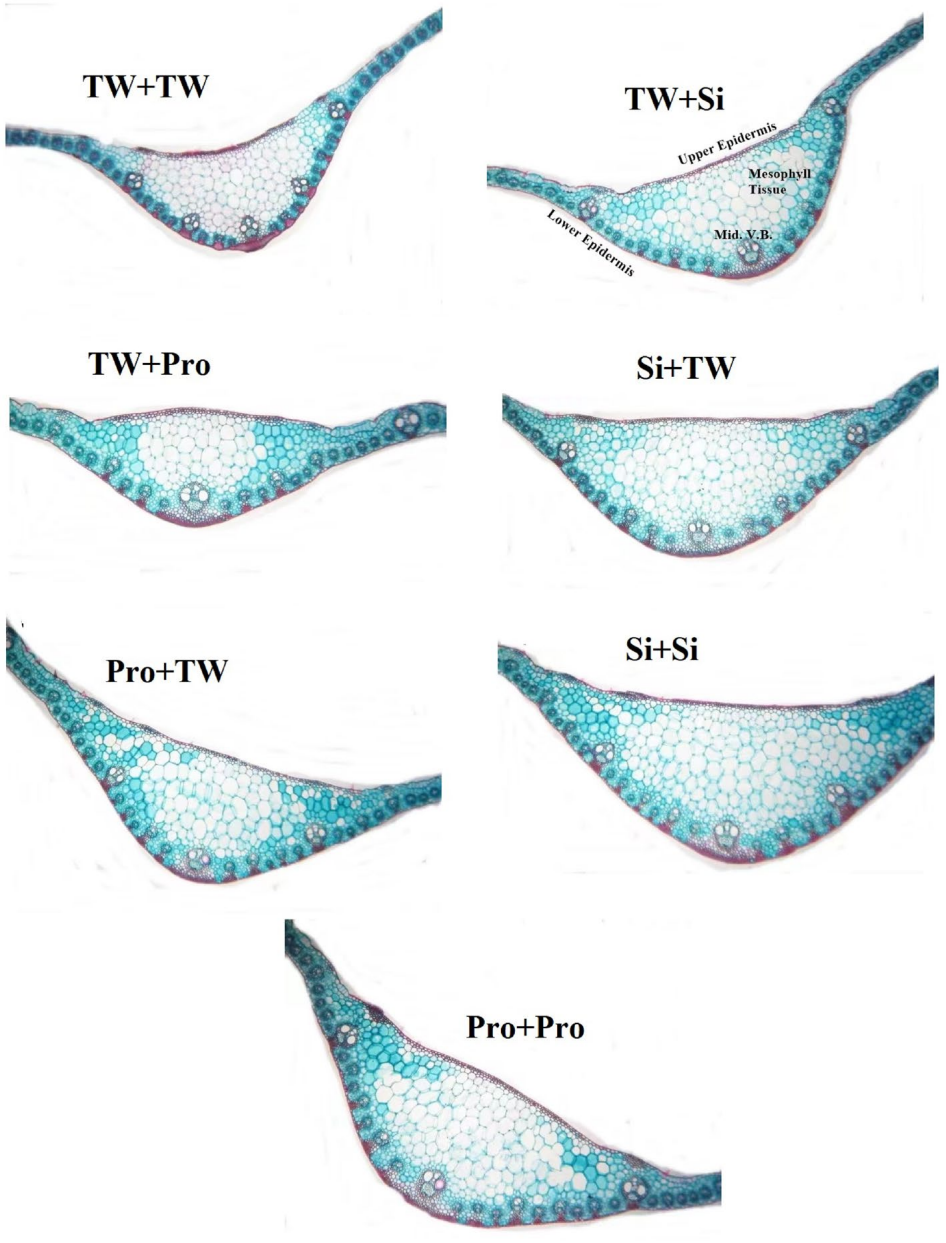
Transverse sections in the flag leaf blade on the main maize stem as affected by foliar application of silicon or proline under salt stress. *Tw* + *TW* seed soaking in and foliar spray with tap water, *TW* + *Si* seed soaking in tap water and foliar spray with silicon, *TW* + *Pro* seed soaking in tap water and foliar spray with proline, *Si* + *Tw* seed soaking in silicon and foliar spray with tap water, *Pro* + *Tw* seed soaking in proline and foliar spray with tap water, *Si* + *SI* seed soaking in silicon and foliar spray with silicon, *Pro* + *Pro* seed soaking in proline and foliar spray with proline, *Mid V.B.* midrib vascular bund.

In the 2022 season, the combination of SS in Si or FS + Pro with Pro or Si therapy improved leaf structural aspects of salt-stressed maize plants vs the comparison (SS in FS + TW with TW) treatment (Table 3, Fig. 5).

**Table 3:** Measurement in microns of certain light microscopically features of a transverse section through the leaf blade from the fourth leaf blade on maize stem as affected with Seed soaking and/or foliar spray by silicon or proline under saline soil condition during the second season.

Seed soaking	Foliar spray	Blade thickness (µ)	Mesophyll tissue thickness(µ)	Length of midrib (µ)	Width of midrib (µ)	Length of midrib vascular bund(µ)	Width of midrib vascular bund(µ)	Diameter of vessel average(µ)
TW	TW	159.6 ± 7f.	106.4 ± 5f.	946.9 ± 14f.	1090 ± 11^g^	106.4 ± 3f.	159.6 ± 5f.	11.90 ± 1.2^d^
TW	Si	212.8 ± 8^d^	127.6 ± 4^d^	1298 ± 16^d^	1254 ± 13^e^	255.3 ± 5^d^	234.0 ± 4^d^	14.28 ± 1.3^c^
TW	Pro	191.5 ± 7^e^	117.1 ± 6^e^	1181 ± 13^e^	1181 ± 16f.	223.4 ± 4^e^	223.4 ± 6^e^	14.28 ± 1.6^c^
Si	TW	223.4 ± 9^c^	148.9 ± 7^c^	1383 ± 15^c^	1999 ± 18^c^	266.0 ± 6^c^	244.7 ± 9^c^	14.28 ± 1.5^c^
Pro	TW	223.4 ± 10^c^	148.9 ± 8^c^	1383 ± 18^c^	1908 ± 19^d^	266.0 ± 7^c^	234.0 ± 6^d^	14.28 ± 1.8^c^
Si	Si	297.9 ± 8^a^	244.7 ± 9^a^	1500 ± 19^b^	2727 ± 22^b^	276.6 ± 5^b^	266.0 ± 8^b^	16.66 ± 1.8^b^
Pro	Pro	234.1 ± 9^b^	191.58^b^	1776 ± 18^a^	3181 ± 26^a^	319.2 ± 9^a^	276.6 ± 9^a^	21.42 ± 2.3^a^

Data are means (n = 5) ± SE. The same letters in each column indicate no significant differences according to the LSD test (p ≤ 0.05).

*TW* tap water, *Si* silicon, *Pro.* proline.

When compared to controls, this treatment dramatically enhanced mesophyll tissue thickness, blade thickness, midrib length, midrib breadth, midrib vascular bund length, midrib vascular bund width, and vessel average diameter.

## Discussion

Salinity in the soil made it difficult for plant roots to get water, which had bad results on the water quality. High respiration rates are linked to metabolic disorders because they hurt meristematic activity and cell elongation, which hinder plant growth and productivity in salty soil^56,57^.

Plants produce ROS, such as superoxide (O_2_^⋅−^), H_2_O_2_, and hydroxyl radicals (OH^−^), in response to salinity stress^3,58–60^. Because ROS damage protein, DNA, membrane functions, and chlorophyll, they produce oxidative stress in plants^61–63^. The plants have developed a sophisticated antioxidant defense mechanism to address and mitigate the negative effects brought on by ROS^64,65^. Proline, carotenoids, and ascorbic acid are only a few of the low-molecular-weight compounds found in plant antioxidant systems^66,67^. According to the structural data available from this study, the negative effect of soil salinity produced a reduction in plant development and yield. This is attributed to osmotic pressure's impact induced by salt stress, which increases growth blockers and decreases growth stimulators^68^. Salinity harms photosynthesis, stomatal closure, ionic and gas exchange imbalances, toxic ion buildup, and growth suppression^11,69^. The current research discovered that using silicon and proline as FS and/or SS significantly enhanced salt-stressed (EC = 7.14 dS m^−1^) maize plant development and output (Table 2), physio-biochemical characteristics (Table 2, Fig. 1), antioxidant enzyme activities (Figs. 3, 4), and leaf anatomical features (Table 3, Fig. 5), when vs the comparison (FS + SS with TW). The integrated Si or Pro SS + FS therapy outperformed all other therapies, consisting of the control (SS in FS + TW with Si or Pro, SS in Si or Pro + FS with TW, and FS + SS with TW).

According to a prior study, the addition of Si significantly improves development characteristics under stressful conditions^70^. Si enhances the growth and productivity of a range of plants by increasing their ability to withstand abiotic stress^71^. Exogenous Si's ability to change the metabolism of cell walls by increasing tissue extensibility and enhancing cell physio-biochemical process activities was discovered to improve bean plant dry weight under various stress conditions by Alzahrani et al.^26^. Si may also improve leaf rigidity by roughening the texture of the leaf^71^. Si is essential for plant growth because of its positive effects on mechanical strength and mineral nutrition, which increases plant resilience to abiotic stressors^72^.

According to Tahir et al.^73^ and other reports^74,75^, exogenous Si has been shown to improve bean plant dry weight under various stress conditions because of its ability to alter cell wall metabolism by boosting cell physio-biochemical process activities and increasing tissue extensibility. Si may also cause a leaf's texture to become rougher, increasing the rigidity of the leaf^72^. According to earlier studies^76,77^, Pro increases growth and keeps nutritional equilibrium in bean plants under stress enhancing P, N, and K absorption.

Many abiotically stressed plants exhibit pro accumulation, a normal physiological response that shows the plant can tolerate the stress. It protects plants from stress by stabilizing membranes, proteins, mitochondrial electron transport complex II, and Rubisco-like enzymes^77,78^. While providing photoassimilates from sappy leaves, maintaining the number of leaves per plant and the amount of green leaf area boosted leaf photosynthesis and increased sink capacity. The results match those published by Thomas and Howarth^79^ exactly.

According to Rios et al.^80^, salt stress causes osmotic stress, which results in nutritional imbalance, toxicity, and oxidative damage, which hinders cellular physiological processes, including photosynthesis. Stress causes the chlorophyllase enzyme to break down chlorophylls, which significantly reduces the amount of photosynthetic pigments^81^.

PSII functional activity can be used to estimate a plant's ability to photosynthesize. According to the study, lower photosynthetic production occurs under salt stress due to functional and structural damage to electron carriers and photosystems. The structural integrity of PSII is harmed by prolonged exposure to high salt stress^82^. In contrast, Si and/or Pro increased the quantity of chlorophyll and the photosynthetic activity in maize plants that were experiencing salt stress in this research (Table 2). In research by Fadzilla and Burdon^83^, Si enhances leaf turgidity by holding the leaf with greater horizontality, which slows down leaf aging, increases chlorophyll content, and increases ribulose-bisphosphate carboxylase activity. Additionally, it improves leaf uprightness to enable enhanced water consumption efficiency (WUE) and leaf water potential as well as light penetration for increased photosynthesis^84^. Si also expands the surface area of the leaf and prevents chlorophyll degradation, allowing for more light to reach the leaves and for photosynthetic activity to take place^85^.

Furthermore, exogenous Si strengthens plant resilience to water stress, according to Ouzounidou et al.^71^. This is because Si paves the path to a steady providing assimilates to developing tissues by maintaining water for developing plant leaf chlorophyll and carotenoids. Si improves water efficiency by contributing significantly to photosynthesis in a variety of abiotic stress circumstances^26^. The reduction of stress-induced damage may be associated with an improvement in the photosynthetic process. According to Ming et al.^86^, Si addition strengthens antioxidant defenses, which lessens oxidative damage to photosynthetic enzymes.

RWC brought on by stress reduces water flow from roots to shoots, which is bad for maize plants. However, water consumption increased when Si or Pro were added to saline soil. Si or Pro increased relative water content, which matched changes in water consumption. It is possible to sustain metabolic activity by making osmotic adjustments and other adaptations to salinity and/or heavy metals when RWC in cells and tissues is in great condition^87^.

Salinized soil in this case reduced RWC, however Si or Pro supplementation may have reduced water stress by increasing WUE. By boosting K translocation and absorption to stomatal protection cells, where K affects the conductivity of the stomata, as well as by decreasing transpiration rate through Si or Pro deposition in leaf and stem epidermis cells, the researchers found that Si or Pro treatments increased RWC under stressful conditions^88^. Si or Pro can advance plant water content when exposed to salt stress, according to research showing that salt stress decreased tomato leaves' osmotic potential (making it more negative) and raised their turgor pressure^89^.

In this research, the RWC of maize plants that have been treated with Si-NPs is superior to that of the control plants. This result is compatible with past findings showing that adding Si or Pro nutrition completely restored RWC to non-stress levels and RWC in wheat plants was much higher than decreased in challenging circumstances^73^.

According to the current research, saline soil stress dramatically decreased MSI. In agreement with Merwad et al.'s^70^ findings, we found results that indicated stress decreased MSI. Si treatment decreased the permeability of the leaf cells' plasma membrane, resulting in improved MSI. An assistive mechanism may be provided by increased MSI after Si therapy because of increased antioxidant activity. According to Agarie et al.^85^, Si enhanced plant growth and production by preserving the membrane integrity and functions of plants under stress from a water shortage. Si enhanced the structural and functional integrity of cell membranes in plants under water deprivation stress, which may have reduced the structural and functional degeneration of cell membranes in stressed plants.

Another mechanism of Si for stress tolerance was suggested by Liu et al.^75^, who found that Si application increased water uptake and transport in sorghum seedlings under stress through the increase of root hydraulic conductance, which attributed to Si-mediated up-regulating the transcription of some aquaporin genes. Additionally, it has been proposed that Si deposition in rice leaves may limit transpiration via the cuticle to preserve plant water content under stressful circumstances, but Si application in maize drastically reduces stomatal transpiration. The mechanical/physical barrier created by Si deposition on these plant surfaces may have had a direct or indirect impact on the plants' capacity to withstand water shortages^74,90^. The higher plant sections were more hydrated as a result of Si treatment, which increased plants' ability to absorb and retain more water.

MDA concentration, which was used to measure lipid peroxidation, is thought to be a good biochemical indication of stress sensitivity and tolerance^91^. When maize seedlings were exposed to salt stress, the MDA concentration increased and was related to oxidative stress (O_2_^−^ and H_2_O_2_). Electrolyte leakage (EL), membrane integrity, and cellular water content are all negatively correlated with higher MDA and oxidative stress^19^. These opposed qualities have a deleterious impact on metabolic activities and, as a result, plant biomass output. In stressed maize plants, the rate of photosynthesis is lowered by shutting the stomata, resulting in a drop in CO_2_ fixation but electron exchange and light response normally continue. Additionally, NADP's ability to absorb electrons is diminished, and oxygen has the potential to serve as an electron receptor. More ROS, such as the 1O_2_, O_2_^⋅−^, H_2_O_2_, and OH^⋅^ radicals that cause cell membrane peroxidation and increase EL, are therefore created^92^.

MDA levels were significantly lowered in stressed maize that had been given Si or Pro treatment. El also comprises. This undesirable outcome was addressed by the interaction of Si and Pro, which offered a superior membrane state than either Si or Pro alone. MDA, a lipid peroxidation byproduct that is found in plant tissue and is assumed to represent the impacts of stress, is reduced by Si. In accordance with the findings of Coskun et al.^93^, Si may therefore help to maintain cell membrane safety while lowering their permeability. The interaction between Si and Pro supports the finding in this study that Si decreases the permeability of the leaf cell plasma membrane by increasing MSI and lowering EL. This positive outcome could be attributed to notable increases in antioxidant activity, which are important defense mechanisms against a range of environmental stressors. Soylemezoglu et al.^94^ found that Si reduces (MDA) concentration and EL as a result of lipid peroxidation in maize and grapevine rootstock.

With or without Si or Pro treatment, the amount of pro and soluble sugars significantly increased in salty soil, acting as an osmoprotective mechanism. A non-enzymatic antioxidant called proline, which rises in concentration in response to stress strengthens plant antioxidant defenses while simultaneously making up for lost plant energy^16,66^. Pro is crucial for the osmotic correction of stressed plant cells^95^. It decreases ROS damage and raises plant tolerance by reducing the detoxification of ROS brought on by salt stress^96^.

Similar to this, the buildup of soluble sugars preserves the proportionality of the proline-like vacuoles and the osmotic properties of the cytosol^47^. A more effective strategy that aids in preserving the status of plant cell water may be the accumulation of suitable solutes under stressful environments. Pro aggregation in salt stress circumstances may be accompanied by a reduction in the rate of proline's oxidative conversion to glutamate, a rise in the proline's enzymatic production, and an increase in its utilization in protein synthesis^97^.

The increase in proline content in plants treated with Si or Pro compared to the control could be ascribed to the proline biosynthesis pathway being upregulated to maintain high levels^26^. The increase in soluble sugar concentration after Si or Pro administration suggests that these compounds have positive effects on the enzymes responsible for carbohydrate metabolism. Additionally, during osmotic stress, Si or Pro enhances the proline pool size and accumulates total free amino acids^70^. According to Rizwan et al.^98^, exogenously administered Si may enhance plant tolerance to drought stress by boosting osmolyte levels and modifying osmotic potential.

In plants growing in salty soil, Si or Pro treatment increased the accumulation of Na + ions and lowered the levels of N, P, Ca, and K ions as well as the K^+^/Na^+^ ratio. This favorable nutritional state was mostly due to silicon's role in improving food absorption. In our investigation, high salt concentrations (EC = 7.11–7.17 dS m^−1^) affect food intake as well as the synthesis and operation of cell membranes. The degree of membrane vector activity, which is involved in the transportation of ions from the soil to the plant and subsequently controls their distribution inside and across plant cells, is a crucial element in defining the nutritional homeostasis of plants^99^.

Due to the Na^+^ ion's antagonistic behavior towards nutrients (i.e. N, K, Ca, and P), membranes can trigger chemical changes in stressed plant cells that represent deficient symptoms in plants^100^. The Si or Pro application, which corrected the K^+^ imbalance found in cells' cytosols on the act of salt stress, positively updated the results of the present study by keeping the cytosolic K^+^ concentrations high and the K^+^/Na^+^ ratio in the proper range as an underlying defense against salt stress in plants, according to Rios et al.^80^. We discovered that treatment with Si or Pro significantly increased the maize's selectivity for nutrient translocation and absorption of N, P, K, and Ca. Supplemental Si or Pro significantly increased the N, P, K, and Ca levels in cucumber plant shoots under salt stress. Si and Pro decreased the osmotic pressure of soil solution that stressors might cause, increasing plant roots' capacity to absorb water and nutrients^19^.

In this research, salt stress-induced oxidative stress led to increased GR activities, SOD, APX, POX, and CAT. High enzymatic activity levels were accelerated and drove them to their peak activity by the interaction between Pro and Si to lessen cellular ROS while under the act of stress. SOD converts stress-related O_2_^⋅−^ generated in plant tissues into H_2_O_2_, which is a potent oxidant because it is produced by SOD canalization. It is forbidden by the glutathione (GSH) and ascorbate (AsA) cycles. The oxidant OH^−^ is also dangerous and extremely reactive. Without restriction, it may respond with any macromolecule. By integrating their behaviors, SOD and CAT can stop or lessen the production of OH^−^^101,102^.

Gong et al.^84^ assert that Si increases CAT and SOD activity. In a different experiment, wheat leaves treated with Si under salt stress exhibited much higher CAT and SOD activity. Because peroxidases (POXs) act by consuming H_2_O_2_ during physiological processes, they can regulate the levels of ROS. POXs have a higher affinity for H_2_O_2_ than CAT, but they can also help oxidize other molecules, such as NADPH, to create H_2_O_2_^103^. Additionally, Li et al.^104^ discovered that POX activity was elevated in salt-stressed Glycyrrhiza uralensis seedlings with 6 mM Si. Si also inhibits the production of ROS and increases its scavenging by antioxidants, both non-enzymatic and enzymatic^80^.

In the current study, salt stress led to an increase in POX activity. By successfully utilizing Si in metabolic pathways that scavenge ROS, Si can therefore improve the safety and integrity of cell membranes at the cellular level and alleviate stress-induced oxidative stress. Additionally, by adding Si and/or Pro to the leaves, maize tolerance to salt stress is boosted, increasing the activity of both enzymatic and non-enzymatic antioxidant mechanisms. In contrast to antioxidant defense systems created with individual Si or Pro application, those given by the interaction between Si and Pro improved maize growth, yield, photosynthetic efficiency, and nutritional homeostasis.

Under typical circumstances, defense mechanisms in plants, such as the buildup of antioxidant molecules, regulate the number of free radicals. To protect stressed plants from oxidative stress caused by salt, soluble sugar, proline, and antioxidant-related chemicals such phenolic, α-TOC, GSH, AsA, and enzymatic antioxidants are biosynthesized and accumulate under salt stress^105^. According to the results of the current study, the integrative application of Si or Pro increased the levels of AsA, α-TOC, and GSH in salt-stressed maize plants, pointing to an improved AsA-GSH cycle, which is crucial for wheat tolerance and the detoxification of ROS^106^.

Additionally, elevated GSH levels support the formation of complexes with phytochelatin, which aid in phytochelatin production and Na ion sequestration in the vacuole^107^. In the present research, Si or Pro treatment enhanced the salt-stressed maize plants' leaf morphological characteristics (Fig. 1), proving that Si or Pro therapy mitigates the negative salt stress's impact on the architecture of the leaf. In the act of challenging salt stress conditions, these enhancements in the anatomical features of the leaf made by Si or Pro enabled a decent relocation of assimilated nutrients entering cells, as well as nutrients for use in various metabolic activities, which are indicative of healthy development and acceptable production. By promoting the development of protective tissue, Si or Pro can be utilized to improve a maize leaf's resistance to dehydration.

## Conclusion

The Si or Pro can be utilized as an effective natural bio-stimulant in maize plants growing under the act of salt stress (EC = 7.14 dS m^−1^) to increase salt tolerance. Si or Pro can be applied as seed soaking to supplement seeds with extra bioactive ingredients (such as AsA, GSH, α-TOC, mineral nutrients, and sugar) for quick and powerful germination. For seedlings to grow quickly and strongly and to promote plant salt tolerance, Si or Pro can also be applied as a foliar spray (integrative application). This can aid plants in surviving in salty soil. The application of Si or Pro, particularly as seed soaking combined with foliar spray and as the best remedy, is found to be effective in reducing salt stress in maize because of the enhanced antioxidant systems; enzymatic antioxidants and non-enzymatic (i.e. GR, APX, SOD, POD, CAT, carotenoids and free proline) and the decreased ROS; O_2_^⋅−^ radicals and H_2_O_2_. The effect caused by Si or Pro is sometimes referred to in this study as the "stay green effect" since its bioactive components enhance the antioxidant defense system in plants under the act of salt stress.

### Supplementary Information

Supplementary Tables.

## Data Availability

The original contributions presented in the study are included in the article/Supplementary Material. Further inquiries can be directed to the corresponding authors.

## References

[1] El-Sappah, A. H., & Rather, S. A. Genomics approaches to study abiotic stress tolerance in plants. *Plant Abiotic Stress Physiol.* **2**, 25–46 (2022).

[2] Munns R (2002). Comparative physiology of salt and water stress. Plant Cell Environ..

[3] El-Sappah, A. H., *et al. *Genome-wide identification and expression analysis of metal tolerance protein gene family in *Medicago truncatula* under a broad range of heavy metal stress. *Front. Genet.* **12**, 713224. 10.3389/fgene.2021.713224 (2021). 10.3389/fgene.2021.713224 PMC8482800 34603378

[4] El-Sappah A. H., *et al. *Interplay of silymarin and clove fruit extract effectively enhances cadmium stress tolerance in wheat (*Triticum aestivum*). *Front. Plant Sci.* **14**(14), 1144319. 10.3389/fpls.2023.1144319 (2023). 10.3389/fpls.2023.1144319 PMC10140571 37123831

[5] El-Sappah A. H., *et al. *Genome-wide identification and expression analysis of metal tolerance protein (MTP) gene family in soybean (*Glycine max)* under heavy metal stress. *Mol Biol Rep.* **50**(4), 2975–2990. 10.1007/s11033-022-08100-x (2023). 10.1007/s11033-022-08100-x 36653731

[6] Mittler R (2002). Oxidative stress, antioxidants and stress tolerance. Trends Plant Sci..

[7] Foyer CH, Noctor G (2000). Oxygen processing in photosynthesis: A molecular approach. New Phytol..

[8] Marschner, H. Mineral nutrition of higher plants, 2nd Ed., Academic Press, London ISBN 01247 35436 (1995).

[9] Rady MM, Desoky EM, Elrys AS, Boghdady MS (2019). Can licorice root extract be used as an effective natural biostimulant for salt-stressed common bean plants?. S. Afr. J. Bot..

[10] Bethke PC, Drew MC (1992). Stomatal and nonstomatal components to inhibition of photosynthesis in leaves of *Capsicum annum* during progressive exposure to NaCl salinity. Plant Physiol..

[11] Rady MM (2003). E. M. Spirulina platensis extract improves the production and defenses of the common bean grown in a heavy metals-contaminated saline soil. J. Environ. Sci..

[12] Kahrizi S, Sedghi M, Sofalian O (2012). Effect of salt stress on proline and activity of antioxidant enzymes in ten durum wheat cultivars. Ann. Biol. Res..

[13] Sitohy MZ, Desoky EM, Osman A, Rady MM (2020). Pumpkin seed protein hydrolysate treatment alleviates salt stress effects on *Phaseolus vulgaris* by elevating antioxidant capacity and recovering ion homeostasis. Sci. Hortic..

[14] FAO. Food and Agriculture Organization of the United Nations; Statistical Database; FAO Rome (2020).

[15] Chinnusamy V, Jagendorf A, Zhu JK (2005). Understanding and improving salt tolerance in plants. Crop Sci..

[16] Desoky EM, Mansour E, Yasin MAT, El-Sobky EEA, Rady MM (2020). Improvement of drought tolerance in five different cultivars of *Vicia faba* with foliar application of ascorbic acid or silicon. Span. J. Agric. Res..

[17] Shirinbayan S, Khosravi H, Malakouti MJ (2019). Alleviation of drought stress in maize (*Zea mays*) by inoculation with Azotobacter strains isolated from semi-arid regions. Appl. Soil Ecol..

[18] Ashraf M, Akram NA (2009). Improving salinity tolerance of plants through conventional breeding and genetic engineering: An analytical comparison. Biotechnol. Adv..

[19] Rady MM, Elrys AS, Abo El-Maati MF, Desoky EM (2019). Interplaying roles of silicon and proline effectively improve salt and cadmium stress tolerance in *Phaseolus vulgaris* plant. Plant Physiol. Biochem..

[20] Qirat M, Shahbaz M, Perveen S (2018). Beneficial role of foliar-applied proline on carrot (*Daucus carota* L.) under saline conditions. Pak. J. Bot..

[21] Yang SL, Lan SS, Gong M (2009). Hydrogen peroxide-induced proline and metabolic pathway of its accumulation in maize seedlings. J. Plant Physiol..

[22] Ihtisham M, *et al*. Primary plant nutrients modulate the reactive oxygen species metabolism and mitigate the impact of cold stress in overseeded perennial ryegrass. *Front Plant Sci.**31*(14), 1149832. 10.3389/fpls.2023.1149832 (2023). 10.3389/fpls.2023.1149832 PMC10103648 37063220

[23] Yildiz M, Terz H (2013). Effect of NaCl stress on chlorophyll biosynthesis, proline, lipid peroxidation and antioxidative enzymes in leaves of salt-tolerant and salt sensitive barley cultivars. J. Agric. Sci..

[24] Singh, M., Kumar, J., Singh, V.P., Prasad, S.M. Proline and salinity tolerance in plants. *Biochem. Pharmacol.* **3**, 6. 10.4172/2167-0501.1000e170 (2014).

[25] Aslam M (2017). Specific role of proline against heavy metals toxicity in plants. Int. J. Pure App. Biosci..

[26] Alzahrani Y, Kuşvuran A, Alharby HF, Kuşvuran S, Rady MM (2018). The defensive role of silicon in wheat against stress conditions induced by drought, salinity or cadmium. Ecotoxicol. Environ. Saf..

[27] Parveen N, Ashraf M (2010). Role of silicon in mitigating the adverse effects of salt stress on growth and photosynthetic attributes of two maize (*Zea mays* L.) cultivars grown hydroponically. Pak. J. Bot..

[28] Shi Y (2016). Silicon enhances water stress tolerance by improving root hydraulic conductance in *Solanum lycopersicum* L. Front. Plant Sci..

[29] Hajiboland R, Cherghvareh L, Dashtebani F (2016). Effect of silicon supplementation on wheat plants under salt stress. J. Plant Process Funct..

[30] Zhang, C.H., Wang, L., Nie, Q., Zhang, W., & Zhang, F. Long-term effects of exogenous silicon on cadmium translocation and toxicity in rice (*Oryza sativa* L.). *Environ. Exp. Bot.* **62**, 300–307 (2008).

[31] Black CA (1968). Soil plant relationships.

[32] Jackson ML (1973). Soil Chemical Analysis.

[33] Dahnke, W. C., & Whitney, D. A. Measurement of soil salinity. In Dahnke, W.C. (Ed.), *Recommended Chemical Soil Test Procedures for the North Central Region*, 499. North Central Regional Publication 221. North Dakota Agricultural Experiment Station Bulletin, pp. 32–34 (1988).

[34] Avron, M. Photophosphorylation by swiss-chard chloroplasts. *Biochim. Biophys. Acta BBA—Ioenerg*. **40**, 257–272 (1960). 10.1016/0006-3002(60)91350-0 13795285

[35] Barrs HD, Weatherley PE (1962). A re-examination of the relative turgidity technique for estimating water deficits in leaves. Aust. J. Biol. Sci..

[36] Premchandra GS, Saneoka H, Ogata S (1990). Cell membrane stability, an indicator of drought tolerance as affected by applied nitrogen in soybean. J. Agric. Sci..

[37] Heath RL, Packer L (1968). Photo peroxidation isolated chloroplasts: Kinetics and stoichiometry of fatty acid peroxidation. Arch. Biochem. Biophys..

[38] Irigoyen JJ, Emerich DW, Sanchez-Diaz M (1992). Water stress induced changes in the concentrations of proline and total soluble sugars in nodulated alfalfa (*Medicago sativa*) plants. Plant Physiol..

[39] Bates LS, Waldren RP, Teare ID (1973). Rapid determination of free proline for water stress studies. Plant Soil..

[40] Wolf B (1982). A comprehensive system of leaf analyses and its use for diagnosing crop nutrient status. Commun. Soil Sci. Plant Anal..

[41] Lachica M, Aguilar A, Yanez J (1973). Analisis foliar: Métodosutilizadosen la Estacion Experimental del Zaidin. An. Edafol. Agrobiol..

[42] Chapman HD, Pratt FP (1982). Determination of minerals by titration method: Methods of analysis for soils, plants and water.

[43] Watanabe FS, Olsen SR (1965). Test of ascorbic acid method for determine phosphorus in water and NaHCO3 extracts from soil. Soil Sci. Soc. Am. Proc..

[44] Vitoria AP, Lea PJ, Azevado RA (2001). Antioxidant enzymes responses to cadmium in radish tissues. Phytochem..

[45] Chance B, Maehly AC (1955). Assay of catalase and peroxidase. Methods Enzymol..

[46] Fielding JL, Hall JL (1978). A biochemical and cytochemical study of peroxidase activity in roots of *Pisum sativum*. J. Expt. Bot..

[47] Sairam RK, Rao KV, Srivastava GC (2002). Differential response of wheat genotypes to long term salinity stress in relation to oxidative stress, antioxidant activity and osmolyte concentration. Plant Sci..

[48] Rao MV, Paliyath G, Ormrod DP (1996). Ultraviolet-B radiation and ozone-induced biochemical changes in the antioxidant enzymes of *Arabidopsis thaliana*. Plant Physiol..

[49] Kampfenkel K, Van Montagu M (1995). Extraction and determination of ascorbate and dehydroascorbate from plant tissue. Anal. Biochem..

[50] Griffth OW (1980). Determination of glutathione and glutathione disulfide using glutathione reductase and 2 vinyl pyridine. Anal. Biochem..

[51] Konings EJ, Roomans HH, Beljaars PR (1996). Liquid chromatographic determination of tocopherols and tocotrienols in margarine, infant foods, and vegetables. J. AOAC Int..

[52] Velikova V, Yordanov I, Edreva A (2000). Oxidative stress and some antioxidant systems in acid rain-treated bean plants. Plant Sci..

[53] Kubis J (2008). Exogenous spermidine differentially alters activities of some scavenging system enzymes, H2O2 and superoxide radical levels in water-stressed cucumber leaves. J. Plant Physiol..

[54] Paolillo DJ, Zobel RW (2002). The formation of adventitious roots on root axes is a widespread occurrence in field-grown dicotyledonous plants. Am. J. Bot..

[55] Johansen DA (1940). Plant Microtechnique.

[56] Abdul Qados AMS (2015). Effects of salicylic acid on growth, yield and chemical contents of pepper (*Capsicum annuum* L.) plants grown under salt stress conditions. Int. J. Agric. Crop Sci..

[57] Kaydan D, Okut MY (2007). Effects of salicylic acid on the growth and some physiological characters in salt stressed wheat (*Triticum aestivum* L.). TarimBİlimleriDergisi.

[58] El-Sappah, A. H., *et al. *Comprehensive genome wide identification and expression analysis of MTP gene family in tomato (*Solanum lycopersicum*) under multiple heavy metal stress. *Saudi J. Biol. Sci.* **28**, 6946–6956. 10.1016/j.sjbs.2021.07.073 (2021). 10.1016/j.sjbs.2021.07.073 PMC8626246 34866994

[59] Lin CC, Kao CH (2001). Cell wall peroxidase activity, hydrogen peroxide level and NaCl-inhibited root growth of rice seedlings. Plant Soil.

[60] Tsai YC, Hong CY, Liu LF, Kao CH (2004). Relative importance of Na+ and Cl− in NaCl-induced antioxidant systems in roots of rice seedlings. Physiol. Plant.

[61] Abbas M, *et al.* Genome-wide analysis and expression profiling of the SlHsp70 gene family in *Solanum lycopersicum* revealed higher expression of SlHsp70–11 in roots under Cd(2+) stress. *Front. Biosci.* (Landmark edition) **27**, 186 (2022). 10.31083/j.fbl2706186 35748262

[62] Cheeseman J (2007). Hydrogen peroxide and plant stress: A challenging relationship. Plant Stress.

[63] El-Sappah, A. H. *et al.* Natural resistance of tomato plants to Tomato yellow leaf curl virus. *Front. Plant Sci.* **13**, 1081549. 10.3389/fpls.2022.1081549 (2022). 10.3389/fpls.2022.1081549 PMC9807178 36600922

[64] Halliwell B, Gutteridge MJC (2007). Free radicals in biology and medicine.

[65] El-Sappah, A. H. *et al.* Heat Stress-Mediated Constraints in Maize (Zea mays) Production: Challenges and Solutions. *Front. Plant Sci.* **13**, 879366. 10.3389/fpls.2022.879366 (2022). 10.3389/fpls.2022.879366 PMC9125997 35615131

[66] Desoky EM, Elrys AS, Rady MM (2019). Integrative moringa and licorice extracts application improves Capsicum annuum fruit yield and declines its contaminant contents on a heavy metals contaminated saline soil. Ecotoxicol. Environ. Saf..

[67] Schutzendubel A, Polle A (2002). Plant responses to abiotic stresses: Heavy metal induced oxidative stress and protection by mycorrhization. J. Exp. Bot..

[68] Desoky, *et al.* Fennel and ammi seed extracts modulate antioxidant defence system and alleviate salinity stress in cowpea (*Vigna unguiculata*). *Sci. Hortic.* **272**, 109576 (2020).

[69] El-Saadony, M. T., *et al*. Biological silicon nanoparticles improve *Phaseolus vulgaris* L. yield and minimize its contaminant contents on a heavy metals-contaminated saline soil. *J. Environ. Sci.* **106**, 1–14 (2021). 10.1016/j.jes.2021.01.012 34210425

[70] Merwad AMA, Desoky EM, Rady MM (2018). Response of water deficit-stressed *Vigna unguiculata* performances to silicon, proline or methionine foliar application. Sci. Hortic..

[71] Ouzounidou G, Giannakoula A, Ilias I, Zamanidis P (2016). Alleviation of drought and salinity stresses on growth, physiology, biochemistry and quality of two *Cucumis sativus* L. cultivars by Si application. Braz. J. Bot..

[72] Hamayun, M., Sohn, E. Y., Khan, S. A., Shinwari, Z. K., Khan, A. L., Lee, I. J. Silicon alleviates the adverse effects of salinity and drought stress on growth and endogenous plant growth hormones of soybean (*Glycine max* L.). *Pak. J. Bot.* **42**(3), 1713–1722 (2010).

[73] Tahir, M.A., *et al.*. Beneficial effects of silicon in wheat (*Triticum aestivum* L.) under salinity stress. *Pak. J. Bot.* **38**(5), 1715–1722 (2006).

[74] Gao X, Zou C, Wang L, Zhang F (2006). Silicon decreases transpiration rate and conductance from stomata of maize plants. J. Plant Nutr..

[75] Liu P (2014). Aquaporin mediated increase in root hydraulic conductance is involved in silicon-induced improved root water uptake under osmotic stress in *Sorghum bicolor* L. J. Exp. Bot..

[76] Ali Q, Ashraf M, Shahbaz M, Humera H (2008). Ameliorating effect of foliar applied proline on nutrient uptake in water stressed maize (*Zea mays* L.) plants. Pak. J. Bot..

[77] Vicente O, AlHassan M, Boscaiu M, Iqbal N, Nazar R, Khan N (2016). Contribution of osmolyte accumulation to abiotic stress tolerance in wild plants adapted to different stressful environments. Osmolytes and Plants Acclimation to Changing Environment: Emerging Omics Technologies.

[78] McNeil SD, Nuccio ML, Hanson AD (1999). Betaines and related osmoprotectants: Targets for metabolic engineering of stress resistance. Plant Physiol..

[79] Thomas H, Howarth CJ (2000). Five ways to stay green. J. Exp. Bot..

[80] Rios JJ, Martínez-Ballesta MC, Ruiz JM, Blasco B, Carvajal M (2017). Silicon mediated improvement in plant salinity tolerance: the role of aquaporins. Front. Plant Sci..

[81] Reddy MP, Vora AB (1986). Changes in pigment composition, hill reaction activity and saccharide metabolism in bajra (*Pennisetum typhoides* S&H) leaves under NaCl salinity. Photosynthetica.

[82] Kalaji HM, Ukarroum A, Brestic M, Zivcak M, Samborska IA, Cetner MD, Łukasik I, Goltsev V, Ladle RJ (2016). Chlorophyll a fluorescence as a tool to monitor physiological status of plants under abiotic stress conditions. Acta Physiol. Plant..

[83] Fadzilla NM, Burdon RH (1997). Salinity, oxidative stress and antioxidant responses in shoot cultures of rice. J. Exp. Bot..

[84] Gong H, Chen K, Chen G, Wang S, Zhang C (2003). Effects of silicon on growth of wheat under drought. J. Plant Nutr..

[85] Agarie S (1998). Effects of silicon on tolerance to water deficit and heat stress in rice plants (*Oryza sativa* L.) monitored by electrolyte leakage. Plant Prod. Sci..

[86] Ming DF, Pei ZF, Naeem MS, Gong HJ, Zhou WJ (2012). Silicon alleviates PEG-induced water-deficit stress in upland rice seedlings by enhancing osmotic adjustment. J. Agron. Crop Sci..

[87] Slabbert MM, Krüger GHJ (2014). Antioxidant enzyme activity, proline accumulation, leaf area and cell membrane stability in water stressed Amaranthus leaves. S. Afr. J. Bot..

[88] Liang, Y., Nikolic, M., Bélanger, R., Gong, H., & Song, A. *Silicon in agriculture: From theory to practice* p. 235 (Springer, New York, NY, USA, 2015).

[89] Romero-Aranda MR, Jurado O, Cuartero J (2006). Silicon alleviates the deleterious salt effect on tomato plant growth by improving plant water status. J. Plant Physiol..

[90] Matoh T, Murata S, Takahashi E (1991). Effect of silicate application on photosynthesis of rice plants. Jpn. J. Soil Sci. Plant Nutr..

[91] Hernández JA, Almansa MS (2002). Short-term effects of salt stress on antioxidant systems and leaf water relations of pea leaves. Physiol. Plant..

[92] Alireza Y, Aboueshaghi RS, Dehnavi MM, Balouchi H (2014). Effect of micronutrients foliar application on grain qualitative characteristics and some physiological traits of bean (*Phaseolus vulgaris* L.) under drought stress. Indian J. Fund. Appl. Life Sci..

[93] Coskun D, Britto DT, Huynh WQ, Kronzucker HJ (2016). The role of silicon in higher plants under salinity and drought stress. Front. Plant Sci..

[94] Soylemezoglu, G., Demir, K., Inal, A., & Gunes, A. Effect of silicon on antioxidant and stomatal response of two grapevine (*Vitis vinifera* L.) rootstocks grown in boron toxic, saline and boron toxic-saline soil. *Sci. Hortic*. **123**(2), 240–246 (2009).

[95] Zhu J-K (2001). Plant salt tolerance. Trends Plant Sci..

[96] Howladar, S. M. A novel Moringa oleifera leaf extract can mitigate the stress effect of salinity and cadmium in bean (*Phaseolus vulgaris* L.) plants. *Ecotoxicol. Environ. Saf.* **100**, 69–75 (2014). 10.1016/j.ecoenv.2013.11.022 24433793

[97] Claussen W (2005). Proline as a measure of stress in tomato plants. Plant Sci..

[98] Rizwan M (2015). Mechanisms of silicon-mediated alleviation of drought and salt stress in plants: A review. Environ. Sci. Poll. Res..

[99] Epstein, E., & Bloom, A. J. Mineral nutrition of plants, principles and perspectives. 2nd Edn. Sinauer Associates, Sunderland, MA ISBN 97808 78931e 729 (2005).

[100] Grattan SR, Grieve CM, Pessarakli M (1999). Mineral nutrient acquisition and response of plants grown in saline environments. Handbook of Plant and Crop Stress.

[101] Kusvuran, S., & Kiran, S. Antioxidant enzyme activities and abiotic stress tolerance relationship in vegetable crops. In Shanker, A. K. (Ed.), *Abiotic and Biotic Stress in Plants—Recent Advances and Future Perspectives*. Intech, Chitra Shanker, pp. 481–506 (Chapter 21) (2016).

[102] Li J (2023). Zinc nanoparticles (ZnNPs): High-fidelity amelioration in Turnip (*Brassica rapa* L.) production under drought stress. Sustainability..

[103] Ranieri A (2005). Oxidative stress and phytochelatin characterisation in bread wheat exposed to cadmium excess. Plant Physiol. Biochem..

[104] Li YT (2016). Silicon nutrition alleviates the lipid peroxidation and ion imbalance of *Glycyrrhiza uralensis* seedlings under salt stress. Acta Physiol. Plant..

[105] Gill SS, Tuteja N (2010). Reactive oxygen species and antioxidant machinery in abiotic stress tolerance in crop plants. Plant Physiol. Biochem..

[106] Hasanuzzaman M (2019). Regulation of ascorbate-glutathione pathway in mitigating oxidative damage in plants under abiotic stress. Antioxidants.

[107] Elrys AS (2022). Mitigate nitrate contamination in potato tubers and increase nitrogen recovery by combining dicyandiamide, moringa oil and zeolite with nitrogen fertilizer. Ecotoxicol. Environ. Saf..

