# Supplementary material for: Integrative application of silicon and/or proline improves Sweet corn (Zea mays L. saccharata) production and antioxidant defense system under salt stress condition
Source: Sci Rep. 2023 Oct 25;13:18315. doi: 10.1038/s41598-023-45003-8 (PMC10600099; doi:10.1038/s41598-023-45003-8)
Supplement: Supplementary file 1 — Supplementary Tables. [file 41598_2023_45003_MOESM1_ESM.docx]

Supplementary Table 1. Seed soaking in and/or foliar spray by silicon or proline effects on relative water content(RWC), membrane stability index (MSI), electrolyte leakage (EL), malondialdehyde (MDA), proline content, total soluble sugars content, nutrient content (i.e. N, P, K, Ca and Na) and K^+^/ Na^+^ ratio of salt-stressed maize plants in two growing seasons.

| **Seed soaking** | **Foliar spray** | **RWC (%)** | **MSI**  **(%)** | **EL**  **(%)** | **MDA**  **(μmol g1)** | **Proline**  **(µg g -1)** | **Total soluble sugars**  **(mg g‒1 dry weight)** |
| --- | --- | --- | --- | --- | --- | --- | --- |
| **1^st^season** | | | | | | | |
| **TW** | **TW** | 64.0±2.6^f^ | 54.3±2.5^g^ | 14.3±0.96^a^ | 2.56±0.11^a^ | 23.5±1.6^g^ | 17.5±1.1^f^ |
| **TW** | **Si** | 76.9±2.9^d^ | 68.3±2.9^e^ | 8.77±0.79^c^ | 1.79±0.08^c^ | 32.5±1.8^e^ | 24.6±1.2^d^ |
| **TW** | **Pro.** | 73.9±3.6^e^ | 66.2±3.1^f^ | 9.33±0.75^b^ | 2.06±0.12^b^ | 29.2±1.9^f^ | 23.4±1.3^e^ |
| **Si** | **TW** | 79.1±3.8^c^ | 73.8±3.6^c^ | 7.41±0.66^e^ | 1.30±0.13^e^ | 35.9±1.5^c^ | 27.6±1.6^b^ |
| **Pro.** | **TW** | 78.2±3.9^c^ | 71.1±3.8^d^ | 7.91±0.65^d^ | 1.45±0.14^d^ | 34.2±2.3^d^ | 26.6±1.5^c^ |
| **Si** | **Si** | 82.2±3.6^a^ | 78.3±4.1^a^ | 6.85±0.45^g^ | 0.95±0.06^g^ | 37.7±2.7^a^ | 28.8±1.7^a^ |
| **Pro.** | **Pro** | 80.5±4.5^b^ | 76.0±3.3^b^ | 7.11±0.52^f^ | 1.08±0.07^f^ | 36.8±2.6^b^ | 28.5±1.8^a^ |
| **2^nd^ season** | | | | | | | |
| **TW** | **TW** | 64.4±3.6^g^ | 54.6±2.3^g^ | 14.0±1.2^a^ | 2.54±0.13^a^ | 23.8±1.3^g^ | 17.8±1.2^f^ |
| **TW** | **Si** | 77.5±4.2^e^ | 68.7±3.6^e^ | 8.47±0.68^c^ | 1.75±0.08^c^ | 32.7±2.2^e^ | 24.8±1.3^d^ |
| **TW** | **Pro.** | 74.1±4.8^f^ | 66.6±3.9^f^ | 9.21±0.85b | 2.04±0.08^b^ | 29.6±1.9^f^ | 23.7±1.2^e^ |
| **Si** | **TW** | 79.5±4.9^c^ | 74.1±4.1^c^ | 7.29±0.78^de^ | 1.26±0.07^e^ | 36.2±1.5^c^ | 27.8±1.8^b^ |
| **Pro.** | **TW** | 78.5±4.8^d^ | 71.4±4.8^d^ | 7.83±0.65^cd^ | 1.40±0.05^d^ | 34.5±1.3^d^ | 26.8±1.7^c^ |
| **Si** | **Si** | 82.6±4.6^a^ | 79.0±4.6^a^ | 6.76±0.66^e^ | 0.93±0.03^g^ | 38.0±2.7^a^ | 29.0±1.8^a^ |
| **Pro.** | **Pro** | 80.7±4.4^b^ | 76.3±3.9^b^ | 7.07±0.75^e^ | 1.05±0.04^f^ | 37.0±2.6^b^ | 28.8±1.9^a^ |
| **Seed soaking** | **Foliar spray** | **N (%)** | **P (%)** | **K (%)** | **Ca (%)** | **Na (%)** | **K^+^/ Na^+^ ratio** |
| **1^st^ season** | | | | | | | |
| **TW** | **TW** | 1.81±0.04^f^ | 0.37±0.02^e^ | 1.55±0.06^g^ | 1.14±0.09^g^ | 2.24±0.14^a^ | 0.69±0.04^g^ |
| **TW** | **Si** | 2.20±0.06^d^ | 0.50±0.04^d^ | 1.78±0.08^e^ | 1.35±0.11^e^ | 1.91±0.12^c^ | 0.93±0.07^e^ |
| **TW** | **Pro.** | 2.16±0.05^e^ | 0.48±0.03^d^ | 1.71±0.06^f^ | 1.24±0.12^f^ | 1.98±0.13^b^ | 0.86±0.06^f^ |
| **Si** | **TW** | 2.36±0.08^b^ | 0.58±0.05^b^ | 2.03±0.09^c^ | 1.97±0.13^c^ | 1.86±0.11^d^ | 1.09±0.09^c^ |
| **Pro.** | **TW** | 2.30±0.09^c^ | 0.54±0.04^c^ | 1.90±0.07^c^ | 1.70±0.14^d^ | 1.88±0.14^cd^ | 1.01±0.06^d^ |
| **Si** | **Si** | 2.42±0.06^a^ | 0.63±0.05^a^ | 2.17±0.08^a^ | 2.16±0.12^a^ | 1.75±0.11^f^ | 1.23±0.09^a^ |
| **Pro.** | **Pro** | 2.39±0.08a^b^ | 0.59±0.03^b^ | 2.07±0.06^b^ | 2.06±0.14^b^ | 1.82±0.13e | 1.14±0.08^b^ |
| **2^nd^ season** | | | | | | | |
| **TW** | **TW** | 1.84±0.05^f^ | 0.38±0.01^e^ | 1.57±0.05^g^ | 1.15±0.07^g^ | 2.21±0.16^a^ | 0.70±0.06^g^ |
| **TW** | **Si** | 2.23±0.06^d^ | 0.51±0.04^d^ | 1.79±0.09^e^ | 1.37±0.08^e^ | 1.88±0.12^c^ | 0.95±0.08^e^ |
| **TW** | **Pro.** | 2.18±0.06^e^ | 0.49±0.02^d^ | 1.72±0.07^f^ | 1.26±0.06^f^ | 1.95±0.14^b^ | 0.88±0.07^f^ |
| **Si** | **TW** | 2.38±0.08^bc^ | 0.60±0.05^b^ | 2.05±0.11^c^ | 1.99±0.08^c^ | 1.83±0.16^c^ | 1.12±0.06^c^ |
| **Pro.** | **TW** | 2.32±0.09^c^ | 0.56±0.03^c^ | 1.92±0.10^d^ | 1.72±0.06^d^ | 1.85±0.15^c^ | 1.03±0.09^d^ |
| **Si** | **Si** | 2.45±0.07^a^ | 0.66±0.05^a^ | 2.19±0.13^a^ | 2.19±0.11^a^ | 1.69±0.13^d^ | 1.29±0.06^a^ |
| **Pro.** | **Pro** | 2.42±0.09^ab^ | 0.61±0.04^b^ | 2.09±0.16^b^ | 2.08±0.13^b^ | 1.75±0.15^d^ | 1.19±0.09^b^ |

Data are means (n=5) ± SE. The same letters in each column indicate no significant differences according to the LSD test (p≤0.05). TW means tap water; Si means Silicon and Pro. mean Proline

Supplementary Table 2. Seed soaking in and/or foliar spray by silicon or proline effects on antioxidant enzymes, hydrogen peroxide (H_2_O_2_), superoxide radical (O_2_^•-^), α-Tocopherol (α-TOC), Ascorbate (AsA) and glutathione (GSH)of salt-stressed maize plants in two growing seasons.

| **Seed soaking** | **Foliar spray** | **CAT** | **POX** | **APX** | **SOD** | **GR** |
| --- | --- | --- | --- | --- | --- | --- |
|  |  | **…………………… (A_564_ min^−1^ g^−1^ protein) …....................** | | | | |
| **1^st^season** | | | | | | |
| **TW** | **TW** | 60.6±3.6^f^ | 0.84±0.07^g^ | 56.9±2.5^f^ | 3.34±0.25^g^ | 34.1±2.6^g^ |
| **TW** | **Si** | 64.3±3.8^d^ | 1.16±0.11^e^ | 60.9±3.1^d^ | 4.74±0.26^e^ | 45.2±3.6^e^ |
| **TW** | **Pro.** | 62.6±3.4^e^ | 0.93±0.12^f^ | 59.0±3.6^e^ | 7.37±0.36^f^ | 44.1±3.5^f^ |
| **Si** | **TW** | 69.4±3.9^b^ | 1.56±0.16^c^ | 63.0±3.8^c^ | 6.76±0.65^c^ | 48.4±3.7^c^ |
| **Pro.** | **TW** | 67.9±4.1^c^ | 1.33±0.11^d^ | 62.8±3.2^c^ | 6.32±0.65^d^ | 47.5±3.8^d^ |
| **Si** | **Si** | 71.3±4.5^a^ | 1.70±0.15^a^ | 66.1±3.9^a^ | 7.22±0.45^a^ | 51.2±3.6^a^ |
| **Pro.** | **Pro** | 69.5±4.6^b^ | 1.63±0.16^b^ | 65.3±3.6^b^ | 7.03±0.55^b^ | 50.3±3.9^b^ |
| **2^nd^ season** | | | | | | |
| **TW** | **TW** | 60.8±4.2^e^ | 0.87±0.06^f^ | 57.3±3.1^e^ | 3.37±0.15^f^ | 34.3±2.5^g^ |
| **TW** | **Si** | 63.7±4.3^d^ | 1.20±0.12^d^ | 61.3±4.2^c^ | 4.77±0.25^d^ | 45.6±3.7^e^ |
| **TW** | **Pro.** | 62.9±4.9^d^ | 0.96±0.07^e^ | 59.4±4.3^d^ | 4.34±0.26^e^ | 44.5±3.3^f^ |
| **Si** | **TW** | 69.8±4.8^b^ | 1.58±0.13^b^ | 63.3±3.9^b^ | 6.84±0.33^b^ | 48.8±3.6^c^ |
| **Pro.** | **TW** | 68.4±4.6^c^ | 1.37±0.14^c^ | 63.0±4.5^b^ | 6.40±0.54^c^ | 47.7±3.5^d^ |
| **Si** | **Si** | 71.9±4.8^a^ | 1.74±0.14^a^ | 66.4±4.9^a^ | 7.26±0.63^a^ | 51.5±4.1^a^ |
| **Pro.** | **Pro** | 70.1±4.9^b^ | 1.66±0.13^ab^ | 66.1±4.3^a^ | 7.11±0.81^b^ | 50.6±4.2^b^ |
| **Seed soaking** | **Foliar spray** | **H_2_O_2_ (mol g^−1^ FW)** | **O_2_^•−^ (A580 g^−1^ FW)** | ***α*-TOC (µmol g^‒1^ DW)** | AsA (µmol g**^‒^**^1^ FW) | GSH (µmol g**^‒^**^1^ FW) |
| **1^st^ season** | | | | | | |
| **TW** | **TW** | 2.46±0.11^a^ | 0.73±0.06^a^ | 1.90±0.15^f^ | 1.21±0.11^g^ | 0.96±0.07^f^ |
| **TW** | **Si** | 1.88±0.12^b^ | 0.54±0.04^c^ | 2.98±0.21^d^ | 1.46±0.12^e^ | 1.45±0.12^d^ |
| **TW** | **Pro.** | 2.01±0.15^b^ | 0.58±0.03^b^ | 2.85±0.25e | 1.40±0.15^f^ | 1.33±0.13^e^ |
| **Si** | **TW** | 1.68±0.16^bcd^ | 0.49±0.02^d^ | 3.19±0.31^b^ | 1.59±0.13^c^ | 1.67±0.15^b^ |
| **Pro.** | **TW** | 1.80±0.13^bc^ | 0.52±0.03^c^ | 3.10±0.32^c^ | 1.50±0.16^d^ | 1.60±0.16^c^ |
| **Si** | **Si** | 1.36±0.14^d^ | 0.40±0.03^f^ | 3.29±0.36^a^ | 1.73±0.13^a^ | 1.77±0.17^a^ |
| **Pro.** | **Pro** | 1.52±0.15^cd^ | 0.46±0.04^e^ | 3.21±0.32^b^ | 1.67±0.15^b^ | 1.70±0.12^b^ |
| **2^nd^ season** | | | | | | |
| **TW** | **TW** | 2.43±0.6^a^ | 0.71±0.05^a^ | 1.93±0.16^d^ | 1.22±0.14^g^ | 0.98±0.05^f^ |
| **TW** | **Si** | 2.00±0.15^c^ | 0.53±0.03^c^ | 2.87±0.19^bc^ | 1.48±0.16^e^ | 1.47±0.09^d^ |
| **TW** | **Pro.** | 2.11±0.19^b^ | 0.56±0.04^b^ | 2.81±0.12^c^ | 1.42±0.15^f^ | 1.36±0.08^e^ |
| **Si** | **TW** | 1.64±0.17^e^ | 0.48±0.02^d^ | 3.22±0.25^ab^ | 1.65±0.16^c^ | 1.69±0.11^b^ |
| **Pro.** | **TW** | 1.85±0.16^d^ | 0.51±0.03^c^ | 3.0±0.21^abc^ | 1.53±0.19^d^ | 1.61±0.13^c^ |
| **Si** | **Si** | 1.33±0.12^g^ | 0.45±0.03^e^ | 3.30±0.26^a^ | 1.78±0.14^a^ | 1.79±0.14^a^ |
| **Pro.** | **Pro** | 1.49±0.13^f^ | 0.39±0.02^f^ | 3.25±0.28^a^ | 1.68±0.13^b^ | 1.72±0.15^b^ |

Data are means (n=5) ± SE. The same letters in each column indicate no significant differences according to the LSD test (p≤0.05). TW means tap water; Si means Silicon and Pro. mean Proline
